# Essentials of Bacteriology

**Published:** 1892-02

**Authors:** 


ESSENTIALS OF BACTERIOLOGY.—Being a Concise and Systematic Introduction to the Study of Micro-Organisms, for the Use of Students and Practitioners. By M. V. Ball, M. D., Late Resident Physician German Hospital, Philadelphia, etc., etc. With Seventy-Seven Illustrations, some in colors. W. B. Saunders, Philadelphia, 1891.

This is a handy compend of the important subject of bacteria, which Koch and other eminent bacteriologists have done so much to elucidate and render invaluable to the science of medicine and surgery.

The history, classifications, structure and microscopical nature of micrococci-bacilli and spirille are given in detail, while the methods most approved of microscopical examination are fully explained.

The chapters on reproduction, cultivation, growth and the pathological influences of the fungi are comprehensive and instructive, while the department of special bacteriology will be found invaluable in the study of the subject. R. W. W.

